# Supplementary material for: Non-native plant removal and high rainfall years promote post-fire recovery of Artemisia californica in southern California sage scrub
Source: PLoS One. 2021 Jul 22;16(7):e0254398. doi: 10.1371/journal.pone.0254398 (PMC8297819; doi:10.1371/journal.pone.0254398)
Supplement: S1 Table — Columns show the year of germination (spring); total spring rainfall (January to June) for that year (mm); treatment (control or non-native removal); the number of marked seedlings that survived; the number of marked seedlings that died; and the percent survival. Percent survival values were only calculated for years in which at least 20 seedlings were tagged for each treatment. Non-native removal treatments were applied in 2014–2017 but not 2018–2019. For 2018–2019, we denoted treatment as “None”, with the original treatment assignment in parentheses (C = control, R = removal). (DOCX) [file pone.0254398.s001.docx]

**S1 Table. Survival of seedlings from when they were marked in June of their first year to the following June.** Columns show the year of germination (spring); total spring rainfall (January to June) for that year (mm); treatment (control or non-native removal); the number of marked seedlings that survived; the number of marked seedlings that died; and the percent survival. Percent survival values were only calculated for years in which at least 20 seedlings were tagged for each treatment. Non-native removal treatments were applied in 2014-2017 but not 2018-2019. For 2018-2019, we denoted treatment as “None”, with the original treatment assignment in parentheses (C=control, R=removal).

|  |  |  |  |  |  |  |
| --- | --- | --- | --- | --- | --- | --- |
| Year | Spring rain (mm) | Treatment | Surv | Died | Percent survived |  |
| 2014 | 271.8 | Control | 4 | 60 | 6.3 |  |
|  |  | Removal | 19 | 89 | 17.6 |  |
| 2015 | 136.7 | Control | 8 | 216 | 3.6 |  |
|  |  | Removal | 36 | 364 | 9.0 |  |
| 2016 | 219.5 | Control | 1 | 4 | **-** |  |
|  |  | Removal | 8 | 9 | **-** |  |
| 2017 | 398.3 | Control | 0 | 0 | **-** |  |
|  |  | Removal | 1 | 2 | **-** |  |
| 2018 | 221.0 | None (C) | 0 | 3 | **-** |  |
|  |  | None (R) | 1 | 0 | **-** |  |
| 2019 | 588.0 | None (C) | 3 | 20 | 13.0 |  |
|  |  | None (R) | 17 | 141 | 10.8 |  |
|  |  |  |  |  |  |  |
|  |  |  |  |  |  |  |
