# Supplementary material for: Non-native plant removal and high rainfall years promote post-fire recovery of Artemisia californica in southern California sage scrub
Source: PLoS One. 2021 Jul 22;16(7):e0254398. doi: 10.1371/journal.pone.0254398 (PMC8297819; doi:10.1371/journal.pone.0254398)
Supplement: S2 Table — The model specifications are given, with fixed effect predictors abbreviated as Size (log transformed canopy volume in m3), Treat (removal or control treatment), and Rain (rainfall from January through June). Random effects for Plot were included in all models, and random effects for Tag (individual plant identity) in the adult models. Terms that changed from the best-fit model are shown (- for removed, + for added), along with the AIC values. (DOCX) [file pone.0254398.s002.docx]

**S2 Table. Summary of all mixed effects survivorship models tested, for both seedlings and adults.** The model specifications are given, with fixed effect predictors abbreviated as Size (log transformed canopy volume in m^3^), Treat (removal or control treatment), and Rain (rainfall from January through June). Random effects for Plot were included in all models, and random effects for Tag (individual plant identity) in the adult models. Terms that changed from the best-fit model are shown (- for removed, + for added), along with the AIC values.

|  |  |  |  |  |
| --- | --- | --- | --- | --- |
|  |  |  |  |  |
| **Age** | **Model** | **Change** | **AIC** |  |
| Seedling | Surv ~ Treat*Rain + (1\|Plot) | Best fit | 618.1 |  |
| Seedling | Surv ~ Treat + Rain + (1\|Plot) | - Treat * Rain | 621.8 |  |
| Seedling | Surv ~ Rain + (1\|Plot) | - Treat | 626.7 |  |
| Seedling | Surv ~ Treat + (1\|Plot) | - Rain | 632.8 |  |
| Adult | Surv ~ log(Size)*Treat + Rain + (1\|Plot) + (1\|Tag) | Best fit | 321.3 |  |
| Adult | Surv ~ log(Size) *Treat + log(Size)*Rain + (1\|Plot) + (1\|Tag) | + log(Size)*Rain | 323.6 |  |
| Adult | Surv ~ Treat*log(Cvol) + (1\|Plot) + (1\|Tag) | - Rain | 325.7 |  |
| Adult | Surv ~ log(Size) + Treat + Rain + (1\|Plot) + (1\|Tag) | - Treat*log(Size) | 327.5 |  |
| Adult | Surv ~ log(Size)*Rain + (1\|Plot) + (1\|Tag) | - Treat, +Rain*log(Size) | 329 |  |
| Adult | Surv ~ log(Size) + Rain + (1\|Plot) + (1\|Tag) | -Treat | 329.7 |  |
| Adult | Surv ~ log(Size) + (1\|Plot) + (1\|Tag) | - Treat, -Rain | 332.5 |  |
| Adult | Surv ~ log(Size) + Treat + (1\|Plot) + (1\|Tag) | - Rain, - Treat*log(Size) | 334.5 |  |
| Adult | Surv ~ Treat + Rain + (1\|Plot) + (1\|Tag) | - log(Size) | 369.2 |  |
|  |  |  |  |  |
|  |  |  |  |  |
|  |  |  |  |  |
