# Supplementary material for: Non-native plant removal and high rainfall years promote post-fire recovery of Artemisia californica in southern California sage scrub
Source: PLoS One. 2021 Jul 22;16(7):e0254398. doi: 10.1371/journal.pone.0254398 (PMC8297819; doi:10.1371/journal.pone.0254398)
Supplement: S3 Table — The most common non-native forb Erodium spp. was unaffected by the removal treatment and is not included; cover values for Erodium can be found in Table 1. Species are annotated by life history (G = non-native annual grasses, F = non-native annual forbs, SS = non-native subshrubs). (DOCX) [file pone.0254398.s003.docx]

**S3 Table. Percent cover of the most common non-native species in control and removal plots for the first four spring surveys (late March to April) after the October 2013 fire.** The most common non-native forb *Erodium* spp. was unaffected by the removal treatment and is not included; cover values for *Erodium* can be found in Table 1. Species are annotated by life history (G = non-native annual grasses, F = non-native annual forbs, SS= non-native subshrubs).

|  |  |  |  |  |  |  |  |  |  |
| --- | --- | --- | --- | --- | --- | --- | --- | --- | --- |
|  | 2014 |  | 2015 |  | 2016 |  | 2017 |  |  |
| Species | Control | Removal | Control | Removal | Control | Removal | Control | Removal |  |
| *Bromus madritensis* (G) | 2.24 | 0.10 | 17.45 | 9.65 | 42.30 | 30.60 | 40.25 | 34.21 |  |
| *Bromus diandrus* (G) | 9.35 | 0.30 | 19.98 | 2.34 | 10.23 | 0.19 | 0.00 | 0.00 |  |
| *Festuca myuros* (G) | 0.00 | 0.00 | 0.00 | 0.39 | 0.00 | 0.00 | 3.22 | 0.58 |  |
| *Brassica nigra* (F) | 4.28 | 0.30 | 11.40 | 0.97 | 6.92 | 1.07 | 4.68 | 0.78 |  |
| *Centaurea melitensis* (F) | 1.07 | 0.60 | 0.49 | 0.10 | 0.78 | 0.29 | 0.58 | 0.00 |  |
| *Marrubium vulgare* (SS) | 0.19 | 0.00 | 0.58 | 0.00 | 0.68 | 0.00 | 0.78 | 0.00 |  |
|  |  |  |  |  |  |  |  |  |  |
