# Supplementary material for: Non-native plant removal and high rainfall years promote post-fire recovery of Artemisia californica in southern California sage scrub
Source: PLoS One. 2021 Jul 22;16(7):e0254398. doi: 10.1371/journal.pone.0254398 (PMC8297819; doi:10.1371/journal.pone.0254398)
Supplement: S4 Table — Numbers for each treatment represent the total plants across all plots in that treatment (N = 6). The percent of plants in removal plots is also shown for each year. (DOCX) [file pone.0254398.s004.docx]

**S4 Table. Distribution of established (greater than one year old) *Artemisia californica* between control and removal plots over the 7 post-fire study years.** Numbers for each treatment represent the total plants across all plots in that treatment (*N* = 6). The percent of plants in removal plots is also shown for each year.

|  |  |  |  |  |
| --- | --- | --- | --- | --- |
| Year | Control | Removal | Percent removal |  |
| 2014 | 3 | 4 | 57.14 |  |
| 2015 | 23 | 22 | 48.89 |  |
| 2016 | 29 | 113 | 79.58 |  |
| 2017 | 16 | 84 | 84.00 |  |
| 2018 | 17 | 72 | 80.90 |  |
| 2019 | 16 | 70 | 81.40 |  |
| 2020 | 19 | 86 | 81.90 |  |
|  |  |  |  |  |
